# Multiocular defect in the Old English Sheepdog: A canine form of Stickler syndrome type II associated with a missense variant in the collagen-type gene *COL11A1*

**DOI:** 10.1371/journal.pone.0295851

**Published:** 2023-12-28

**Authors:** Katherine Stanbury, Renata Stavinohova, Louise Pettitt, Chris Dixon, Ellen C. Schofield, Bryan Mclaughlin, Inka Pettinen, Hannes Lohi, Sally L. Ricketts, James A. Oliver, Cathryn S. Mellersh

**Affiliations:** 1 Kennel Club Genetics Centre, Department of Veterinary Medicine, University of Cambridge, Cambridge, United Kingdom; 2 Lumbry Park Veterinary Specialists, Hampshire, United Kingdom; 3 Veterinary Vision, Cumbria, United Kingdom; 4 Department of Veterinary Biosciences, Department of Medical and Clinical Genetics, University of Helsinki and Folkhälsan Research Center, Helsinki, Finland; 5 Dick White Referrals, Cambridgeshire, United Kingdom; Mayo Clinic Minnesota, UNITED STATES

## Abstract

Multiocular defect has been described in different canine breeds, including the Old English Sheepdog. Affected dogs typically present with multiple and various ocular abnormalities. We carried out whole genome sequencing on an Old English Sheepdog that had been diagnosed with hereditary cataracts at the age of five and then referred to a board-certified veterinary ophthalmologist due to owner-reported visual deterioration. An ophthalmic assessment revealed that there was bilateral vitreal degeneration, macrophthalmos, and spherophakia in addition to cataracts. Follow-up consultations revealed cataract progression, retinal detachment, uveitis and secondary glaucoma. Whole genome sequence filtered variants private to the case, shared with another Old English Sheepdog genome and predicted to be deleterious were genotyped in an initial cohort of six Old English Sheepdogs (three affected by multiocular defect and three control dogs without evidence of inherited eye disease). Only one of the twenty-two variants segregated correctly with multiocular defect. The variant is a single nucleotide substitution, located in the collagen-type gene *COL11A1*, c.1775T>C, that causes an amino acid change, p.Phe1592Ser. Genotyping of an additional 14 Old English Sheepdogs affected by multiocular defect revealed a dominant mode of inheritance with four cases heterozygous for the variant. Further genotyping of hereditary cataract-affected Old English Sheepdogs revealed segregation of the variant in eight out of nine dogs. In humans, variants in the *COL11A1* gene are associated with Stickler syndrome type II, also dominantly inherited.

## Introduction

Multiocular defect (MOD) is an inherited ocular syndrome that has been described in a number of dog breeds that include Dobermann, Portuguese Water Dog, Akita, Australian Shepherd cross and Cavalier King Charles Spaniel [1–5]. Abnormalities reported vary between breeds, but dogs can be affected with cataracts, microphthalmia, uveitis, coloboma, lenticonus, retinal dysplasia and retinal detachment. A previous report of MOD in the Old English Sheepdog (OES) describes similar clinical characteristics, including congenital/nuclear cataract, retinal folds and microphthalmos [6]. The British Veterinary Association/Kennel Club/International Sheep Dog Society (BVA/KC/ISDS) Eye Scheme is an eye examination scheme, the purpose of which is to ensure that there is no clinical evidence of hereditary eye disease in dogs that are to be used for breeding. MOD was included in the now defunct Schedule B of the Scheme, which listed ocular conditions under investigation due to their likely hereditary nature and has been described as a genetic disease with an unknown mode of inheritance [7]. Hereditary Cataract (HC) is the only eye condition currently certifiable in the OES breed under the BVA/KC/ISDS Eye Scheme [8]. The cataract is described in the literature as bilateral asymmetrical cortical opacities [9] which may progress to total cataract occurring from seven months to two years [10]. In the OES, cataract has been associated with retinal detachments [6, 9, 10] and Barnett (1978) further postulated that cataract in the breed may occur in conjunction with multiple ocular anomalies. Cataract was observed to be the most common phenotype in MOD-affected OES in this study.

Canine MOD shares homologous ocular characteristics with the human hereditary arthro-ophthalmological conditions of Stickler syndrome and Marshall syndrome [11, 12]. Stickler syndrome is now an encompassing term used to describe a group of genetically heterogeneous connective tissue dysplasias [13]. Stickler syndromes type I (STL1) and type II (STL2) are both characterised by features such as vitreopathy, retinal detachment and macrophthalmos, in addition to systemic abnormalities that can include craniofacial abnormalities, deafness and arthropathy. Marshall syndrome has considerable phenotypic overlap with Sticker syndrome. Differentiating factors for Marshall syndrome are distinctive facial features such as saddle nose, upward-turned nostrils and dental malformations [14]. MOD also shares ocular abnormalities described in Samoyed, Labrador Retriever and Northern Inuit Dog canine breeds with oculoskeletal dysplasia (OSD) [15–18]. The canine OSDs are recessively inherited and are associated with a nonsense variant in *COL9A3* in the Northern Inuit [16], a frameshift variant also in *COL9A3* in the Labrador Retriever, and a 1,267bp deletion in *COL9A2* in the Samoyed [18]. OSD is characterised by short-limbed dwarfism and skeletal dysplasia in conjunction with ocular abnormalities including macrophthalmos, vitreopathy, retinal dysplasia, retinal tears/detachment and cataract [16, 18]. There also appears to be a carrier affect in Samoyed and Labrador Retrievers, as dogs heterozygous for their breed-specific OSD variants were described as having retinal folds with incomplete penetrance [18]. The folds in OSD carriers are described as distinct, appearing as clusters and with a specific distribution in the posterior pole [19]. All aforementioned inherited disorders include additional systemic abnormalities that can include oro-facial, musculoskeletal and auditory malformations, with the exception of Stickler syndrome for which ocular-only forms have also been described [20].

To date, no genetic variants have been attributed to cause MOD in the dog. Furthermore, the mode of inheritance appears to be ambiguous. It is postulated to be autosomal recessive in the Dobermann [4] and either recessive or dominant with incomplete penetrance in the Akita [5]. Therefore, to investigate MOD in the OES we selected a whole genome sequencing approach to elucidate the disease’s genetic cause and mode of inheritance for the disease.

The OES is a large herding breed that emerged in England during the nineteenth century, with the breed’s ancestors possibly including the Scottish Collie, Bearded Collie and Ovtcharka. It is a numerically small breed, with the number of dogs registered with the Kennel Club each year declining over the last decade. It is currently listed on the Kennel Club’s Vulnerable ‘At Watch’ list [21].

This study describes the clinical and histopathological findings and genetic basis of MOD in OES.

## Materials and methods

### Sample collection

Dog owners and veterinary ophthalmologists submitted OES DNA samples to the Kennel Club Genetics Centre (previously based at the Animal Health Trust, Newmarket, UK) as buccal mucosal swabs or residual blood samples, with owner consent (AHT Ethics Approval ref: 24-2018E, University of Cambridge Ethics Approval ref: CR564). Unaffected dogs are defined as those certified clear of inherited eye disease under the BVA/KC/ISDS Eye Scheme or European/US equivalent or by a veterinary ophthalmologist (*n* = 48). OES MOD-affected dogs are defined as dogs diagnosed with MOD by a veterinary ophthalmologist (*n* = 13) or dogs diagnosed with two or more phenotypes of MOD as described in Table 1 by a veterinary ophthalmologist (*n* = 4). Nine OES were defined as HC-affected; OES diagnosed with cataract (not HC type) (*n* = 6); or OES diagnosed with an ocular abnormality not certifiable under the BVA/KC/ISDS Eye scheme (*n* = 6). DNA was extracted from both blood and buccal swabs using QIAamp DNA Blood Mini or Midi Kits (Qiagen, Manchester, UK).

**Table 1:** Core phenotypes in MOD-affected OES. Shaded boxes indicate that the phenotype was observed in the case.

	Age of Diagnosis (yrs)	Cataract	Cataract Description	Macrophthalmos	Vitreopathy	Microphakia	Lens Coloboma	Retinal Detachment	Genotype
**Case 1**	0.5		Mature						C/C
**Case 2**	2.33		Cortical, immature						C/C
**Case 3**	2.4		Mature						C/C
**Case 4**	0.67		Nuclear cataract, with diffuse anterior and posterior cortical cataract						C/C
**Case 5**	1.75		Cortical						C/C
**Case 6**	0.75		Anterior and posterior subcapsular cortical with lenticular vacuolation						C/C
**Case 7**	0.75		Anterior and posterior cortical						C/C
Case 8	0.75		Anterior and posterior diffuse cortical						C/C
**Case 9**	0.58								C/C
**Case 10**	5		Subcapsular capsular cortical, immature						C/C
**Case 11**	1.83		Dense nuclear and diffuse cortical						C/C
**Case 12**	3		Cataract (no further description)						C/C
**Case 13**	2.5		Diffuse nucleo-cortical						C/C
**Case 14**	9.7		Cortical immature						T/C
**Case 15**	0.75		Punctate equatorial						T/C
**Case 16**	2.4		Anterior and posterior cortical						T/C
**Case 17**	1.5		Posterior cortical and nuclear						T/C

Additional OES samples (disease status unknown) were collected and genotyped for the *COL11A1* variant (*n* = 93*)* by the University of Helsinki, Finland (Ethics approval: Animal Experimental Board of Regional State Administrative Agency of Southern Finland (ESAVI/7482/04.10.07/2015, ESAVI/25696/2020).

### Ophthalmic investigation

Ophthalmic examination included vision assessment, Schirmer tear test (MSD Animal Health, Milton Keynes, Bucks, UK), slit-lamp biomicroscopy (Kowa SL-17, Torrance, California, USA), rebound tonometry (TonoVetTM iCare, FI-01510 Vantaa, Finland), and direct and indirect ophthalmoscopy (Keeler Professional, Windsor, Surrey, UK), including following pharmacological mydriasis with topical ophthalmic 1% tropicamide eye drops (Minims, Bausch & Lomb, Kingston-upon-Thames, Surrey, UK). B-mode ocular ultrasound was also performed to measure the globe and lens and assess the posterior segment (Easoate MyLabONE, 6–18MHz 30 mm linear probe, Imotek, Somersham, Cambs, UK). Ultrasound transmission gel (Aquasonic, Parker laboratories, USA) was used as a coupling medium. Gonioscopy and/or electroretinography was conducted in cases where the owners opted for possible cataract surgery. Gonioscopy and ultrasound were both performed after instillation of topical anaesthetic 0.5% proxymetacaine eye drops (Minims, Bausch & Lomb, Kingston-upon-Thames, Surrey, UK).

### Histology

Histopathological examination of an enucleated globe of a MOD OES case was performed by Dick White Referrals Diagnostics, Suffolk, UK. The eye was removed for welfare reasons due to non-controlled end-stage secondary glaucoma leading to an irreversibly blind and painful eye.

### Whole genome sequencing

A seven-year-old female OES, confirmed to be a MOD case by a board-certified veterinary ophthalmologist was selected for whole genome sequencing (WGS). Sequencing was outsourced to Edinburgh Genomics, UK where a TruSeq Nano library was prepared and sequenced on the Illumina HiSeq X platform, generating approximately 30X genome coverage. Paired-end sequence data were aligned to the canine reference genome CanFam3.1 using the BWA-MEM algorithm [22] and variant calls were made using GATK v3.6 [23]. A multi-sample Variant Call Format (VCF) file was created by consolidating and joint-calling Genomic Variant Call Format (gVCF) files of 219 canine genomes (inclusive of the OES MOD case). Variant calls were annotated using Variant Effect Predictor (VEP) [24] and visualised in the Integrative Genomics Viewer (IGV) software [25, 26].

### Variant filtering

Variants were primarily filtered against 219 WGS of 102 breeds and five crossbreeds via an in-house pipeline that scores each variant based on the predicted effect on the protein and by its potential pathogenicity with a Sorting Intolerant from Tolerant (SIFT) score [27]. Variants private to the case and predicted to be deleterious were then compared against 613 WGS of 117 dog breeds, eight wolf and 28 Chinese Indigenous dogs as part of the Dog Biomedical Variant Database Consortium (DBVDC) [28]. Genes containing potential candidate variants were cross-referenced with keywords (S1 Table) relating to the MOD condition using the NGS phenotyper software VarElect (Lifemap Sciences). Variants within genes cited to correlate with the MOD phenotype directly were retained for disease segregation analysis.

### Sanger sequencing of filtered variants

Twenty-two variants, one homozygous and 21 heterozygous in the MOD WGS case, were genotyped by Sanger sequencing in a small cohort of three MOD cases and three control OES (MOD unaffected) whose clinical status had been confirmed by a veterinary ophthalmologist. Primers were designed using Primer 3 [29] to flank the variant (S2 Table) and amplified by PCR using HotStarTaq DNA Polymerase (Qiagen) with the following cycling conditions: 98°C for 10 mins; 35 cycles at 98°C for 30s; 57°C (59°C for variant with an asterix) for 30s; 72°C for 30sec and then 72°C for 5 mins. PCR products were sequenced in both directions, at the Department of Biochemistry, University of Cambridge. Sequence traces were analysed using the Staden software package [30].

### Sanger sequencing of *COL11A1* variant

To validate the candidate *COL11A1* c.4775T>C variant, in addition to the preliminary six OES, a further cohort comprising 80 OES and fourteen control dogs of different breeds (S3 Table) was genotyped using Sanger sequencing as above. The 80 OES comprised 45 unaffected dogs, 14 MOD cases, nine HC-affected, six non-HC-cataract affected and six dogs diagnosed with other ocular abnormalities (Table 3).

### Pathogenicity and splicing effects of *COL11A1* variant

Pathogenicity was further evaluated using Polyphen-2 (http://genetics.bwh.harvard.edu/pph2) [31] and UMD Predictor-pro (https://umd-predictor.genomnis.com/mutation) [32]. Splicing signals were analysed with HSF Pro (Genomnis) (https://hsf.genomnis.com/home), UMD Predictor-Pro (https://umd-predictor.genomnis.com/mutation) [32], Ex-Skip (https://ex-skip.img.cas.cz/) [33], Sroogle (http://sroogle.tau.ac.il/) [34] and ESE Finder 3.0 (https://esefinder.ahc.umn.edu/cgi-bin/tools/ESE3/esefinder.cgi) [35, 36].

## Results

### Phenotype of MOD-affected OES

#### Clinical characterisation of MOD cases

Full ophthalmic examination was performed in all 17 MOD cases. Gonioscopy was performed in four cases (1,2,3,10), revealing normal iridocorneal angle and pectinate ligament anatomy. Electroretinography was performed in one MOD case (10) and revealed reduced photoreceptor function (data not available).

The most commonly occurring (core) phenotypes observed in MOD-affected OES are listed in Table 1, with globe, lens, vitreous and retina being most affected. Phenotypes include those observed at initial diagnosis and any follow-up consultations (see S4 Table for further phenotypic details of the 17 cases).

Bilateral macrophthalmos was recorded in 11 MOD cases as shown in Table 1 (*n* = 22 eyes). Ocular ultrasound revealed a mean globe anteroposterior distance of 28.49mm (Table 2), which is 7.59mm longer than a previously reported mean distance of 20.9mm, derived from 22 dogs (*n* = 44 eyes) of 15 different breeds [37]. It should be noted that OES was not included in the previous study.

**Table 2:** Anteroposterior globe lengths recorded in MOD-affected OES.

Case number	OD length (mm)	OS length (mm)
1	25.0	26.0
2	30.8	28.9
3	26.1	28.9
6	27.8	28.0
8	28.6	29.1
11	32.2	31.8
13	27.8	27.8
**Mean**	**28.33**	**28.64**
**SD**	**2.51**	**1.75**
**SEM**	**0.95**	**0.66**

With the exception of one MOD-affected OES, all other MOD cases presented with cataracts. The outlier (Case 9 in Table 1) was examined at 7 months old and diagnosed with macrophthalmos, microphakia and retinal detachment. It was not possible to carry out a follow-up examination on this case to ascertain if cataracts developed after this time, therefore it cannot be formally excluded. In three UK cases (5, 10, 17) the cataract was diagnosed as HC during a BVA/KC/ISDS eye exam and noted as such.

Microphakia was observed in 11 MOD cases (*n* = 22 eyes). Lens coloboma was recorded in eight MOD cases (*n* = 14 eyes). Vitreopathies were noted in nine MOD cases (*n* = 17 eyes). Two cases were noted to have liquified vitreous bilaterally. One case had unilateral anterior vitreal prolapse. Persistent hyaloid artery remnants were recorded in one dog (*n* = 2 eyes). Retinal detachment was observed in 11 dogs (*n* = 18 eyes).

Follow-up reports for five OES MOD cases (1,2,3,10,14) revealed the progression of pathologies. These included, cataract progression and blindness, lens-induced uveitis, lens sub/luxation, retinal detachment and secondary glaucoma. Enucleation was performed in two dogs (2,10) (*n* = 2 eyes). Both underwent unilateral enucleation due to uncontrolled secondary glaucoma associated with chronic lens-induced uveitis.

Phacoemulsification or intracapsular lens extraction was performed in five dogs (8, 10, 11, 13, 14) (n = 6 eyes). Vitreoretinal surgery was performed in two dogs (8,13) (n = 3 eyes).

#### Histology results

Histology of an enucleated globe from an OES diagnosed with macrophthalmos, microphakia, lens coloboma, cataract and partial retinal detachment revealed an enlarged globe (35x35x34mm) and a cataractous change in lens. Microscopic results confirmed a markedly enlarged globe. The retina was detached and degeneration was detected in the outer retinal layers. The retina was folded and exhibited partial loss of layering, with thinning of all layers, nuclear loss and apparent retention of the ganglion cell layer. The lens capsule was detached with lens fibres multifocally replaced by eosinophilic granules and multifocal mineralised foci within the lens cortex. There was a loss of normal architecture of the iridocorneal angle.

### Identification of a collagen type XI alpha 1 chain gene variant associated with MOD in the OES

**Fig 1:**
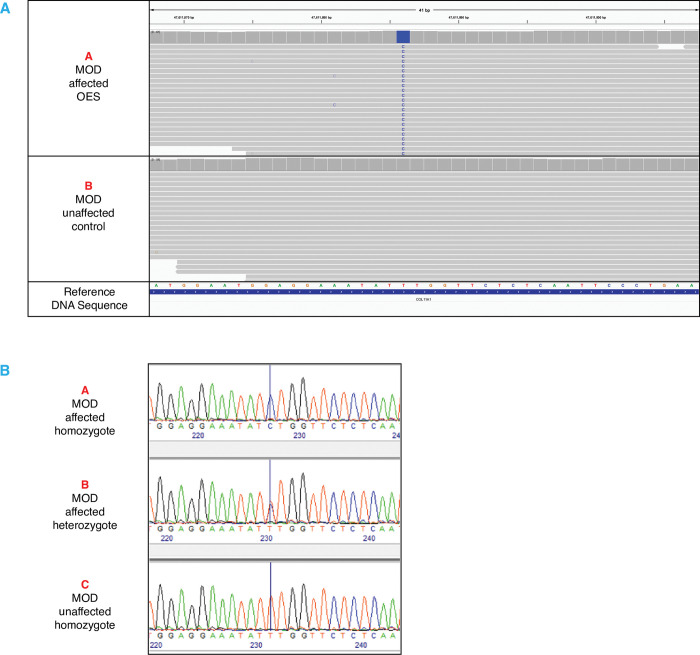
Missense variant identified in *COL11A1*. Illustration of the *COL11A1* T>C variant located at Chr6:47611886 (Canfam 3.1) (**A**) WGS reads in IGV of an MOD affected OES homozygous for the C/C variant and an MOD unaffected control (Border Collie) homozygous for the reference allele T/T. (**B**) Illustrates electropherogram Sanger sequence reads of the variant in two affected MOD OES who are homozygous C/C and heterozygous C/T. The MOD unaffected OES is homozygous T/T for the reference allele.

Whole genome sequence (WGS) from case 10 was compared to the WGS data of 218 dogs of other breeds unaffected by MOD. Seventeen DNA variants were identified as being private to the MOD case and predicted to be deleterious. Five additional variants predicted to be deleterious in the case were shared with an OES within the DBVDC dataset. This dog had died at 1.5 years of age, of a condition unrelated to MOD, and no information regarding the ocular health of this dog was available. All twenty-two variants were investigated. Only one segregated correctly in the cohort of three cases and three controls, a T>C single nucleotide polymorphism (SNP) located in the *COL11A1* gene (Fig 1). The SNP is within an exon present within four of the five *COL11A1* isoforms annotated in canine genome build CanFam3.1 and is a missense variant; c.1775T>C, p.Phe1592Ser (chr6:47,611,886). The amino acid is conserved across 200/201 mammalian species (See S1 Appendix). In addition to the variant predicted to be deleterious by SIFT, PolyPhen-2 calculated the amino acid substitution to be ‘Probably Damaging’ with a score of 0.997 and 100% damaging by UMD Predictor-Pro.

#### Splice signal analyses

The HSF pro web tool (Sequence Analysis) revealed that there is a single donor site (signal score 66.6) adjacent to the MOD variant site and a branch point (signal score 93.38) 6bp downstream. However, the T>C SNP was not predicted to impact splicing signals in the transcripts significantly. In addition to a score of 100% pathogenicity in UMD-Predictor-Pro data points relating to splice elements were lower than threshold data in the radar plot of ‘pathogenicity prediction’ (See S2 Appendix). These related to the exonic splice enhancer (ESE)/exonic splice silencer (ESS) ratio, HSF New acceptor/donor site and HSF Broken acceptor/donor site. The plot also illustrates that the amino acid is conserved as this data point exceeds the threshold value. EX-SKIP, Sroogle and ESE Finder all reported variations between the reference and variant alleles with regard to splice elements. EX-SKIP reported a higher ESS to ESE ratio in the wild-type sequence with 0.26 and 0.23 in the mutant. Similarly, ESE Finder reported a novel enhancer site (SRp40) not present within the wild type sequence and Sroogle an increased density of SRp40 in the mutant sequence compared to the wild type, with 0.17 and 0.14 respectively.

### *COL11A1* variant segregates with disease status amongst MOD cases and most HC-affected OES

All 17 OES MOD-affected dogs (Table 1) were either heterozygous (*n* = 4) or homozygous (*n* = 13) for the *COL11A1* c.4775T>C variant (Table 3). The age of diagnosis for the four heterozygotes ranged from 0.75 to 9.67 years with a median age of 1.95 years. The thirteen homozygotes were diagnosed between the ages of 0.5 to 5 years with a median age of 1.75 years.

**Table 3:** Results of OES breed *COL11A1* genotyping. Distribution of *COL11A1* genotypes in dogs with ocular phenotypes and unaffected dogs.

	MOD- Affected	HC- Affected	Non-HC- cataract	*Other ocular abnormalities	Unaffected	TOTAL
**Homozygous Reference (T/T)**	0	1	4	1	42	**48**
**Heterozygote (T/C)**	4	4	2	4	6	**20**
**Homozygous *COL11A1 variant* (C/C)**	13	4	0	1	0	**18**
**TOTAL**	**17**	**9**	**6**	**6**	**48**	**86**

*Other ocular abnormalities; Pigment in anterior vitreous (*n* = 1); persistent pupillary membranes (*n* = 3); retinal folds (*n* = 1); retinal detachment (*n* = 1). Non-HC cataract; posterior polar subcapsular cataract (*n* = 1); unilateral (*n* = 2); capsular cataracts (*n* = 1); pinpoint lens opacities (*n* = 1); perinuclear+focal peripheral cataracts (*n* = 1).

Eight of the nine HC-affected OES were also homozygous or heterozygous for the *COL11A1* variant, with only one individual homozygous for the reference allele. This dog was diagnosed with congenital HC at 0.17 yrs. Of the four HC cases homozygous for the variant, two were diagnosed at litter screen and the remaining two were diagnosed at 1.5 and 1.25 years. The latter homozygous OES had mature bilateral cortical cataracts upon diagnosis. The four heterozygotes were diagnosed from the ages of 0.17 to 5 years, one of whom had abnormal retinal vessels recorded on the eye certificate.

#### Further *COL11A1* genotyping results

Table 4 shows six OES diagnosed with cataracts that were not typical of HC in the OES breed (listed as non-HC cataract in Table 3). Two had been diagnosed with unilateral cataracts, one aged 6 years and the other 0.58 years. Both of these dogs were homozygous for the reference allele. An OES diagnosed with bilateral posterior polar subcapsular cataract at litter screen and an OES diagnosed at 1.17 years with posterior capsular cataract in the left eye and anterior capsular cataract in the right eye were also both homozygous for the reference allele. The remaining two non-HC type cataract OES were described as; unilateral pin-point lens opacities in one individual at age 0.75 years and bilateral perinuclear and focal peripheral cataracts in the other at age 2.75 years. The latter had a chorioretinal scar in the mid-tapetum in the left eye. Both individuals were heterozygous for the *COL11A1* variant.

**Table 4:** *COL11A1* genotyping results and cataract types of OES diagnosed with non-HC cataract.

Genotype	Age at Diagnosis	Cataract type
**Homozygous Reference (T/T)**	Litter Screen	Posterior polar subcapsular (*n* = 2 eyes)
**Homozygous Reference (T/T)**	0.58	Small, dense cortical (*n* = 1 eye)
**Homozygous Reference (T/T)**	1.17	Anterior capsular cataract (right eye)–Posterior capsular cataract (left eye)
**Homozygous Reference (T/T)**	6.08	Cataract (*n* = 1 eye)
**Heterozygote (T/C)**	4.75	Perinuclear + focal peripheral (*n* = 2 eyes) + left scar mid tapetum
**Heterozygote (T/C)**	0.75	Pinpoint opacities (*n* = 1 eye)

Six OES had been diagnosed (Table 5) with ocular abnormalities that were not certifiable under the BVA/KC/ISDS Eye Scheme in the OES breed, listed as ‘Other Ocular Abnormalities’ in Table 3. Three examined at 0.17 years old were diagnosed with persistent pupillary membranes (PPM), two heterozygous, and the third homozygous, for the reference allele. The remaining two heterozygotes were a 5.5yr old dog with pigment in the anterior vitreous and an OES who presented with retinal folds at age 1.58 years. One dog diagnosed with retinal detachment aged 0.33 years was found to be homozygous for the MOD variant.

**Table 5:** *COL11A1* genotyping results and phenotypes of OES diagnosed with other ocular abnormalities.

Genotype	Age at Diagnosis	Ocular phenotype
**Homozygous Reference (T/T)**	0.17	Hereditary persistent pupillary membranes (*n =* 2 eyes)
**Heterozygote (T/C)**	0.17	Persistent pupillary membranes (*n =* 2 eyes)
**Heterozygote (T/C)**	0.17	Persistent pupillary membranes (*n = 1* eye)
**Heterozygote (T/C)**	5.5	Pigment in anterior vitreous (*n = 1* eye)
**Heterozygote (T/C)**	1.58	Multiple retinal folds of the left fundus (*n = 1* eye)
**Homozygous *COL11A1 variant* (C/C)**	0.33	Retinal detachment (*n =* 2 eyes)

Of the 48 OES dogs that were categorised as unaffected for inherited eye disease, 42 were homozygous for the reference allele (age of examination ranging from eight weeks to 11 years, median age 3.29 years) and six were heterozygous for the *COL11A1* c.4775T>C variant (age of examination ranging from 1.25 to 2.58 years, with a median age of 1.7 years) when their eyes were examined.

An assessment of the allele frequency of the MOD variant in an independent set of OES (Helsinki OES cohort) is shown in Table 6, with four dogs heterozygous for the MOD variant. Follow-up of the four dogs found that they were reported to be clear of inherited eye disease between the ages of 2.1 to 6.8 years old.

**Table 6:** *COL11A1* allele frequencies in the University of Helsinki cohort.

	T/T	T/C	C/C	Total OES Genotyped
	(Reference)	(heterozygote)	(Variant)	
**University of Helsinki**	0.96	0.02	0.00	93

Fourteen dogs of 10 different breeds which were either clear of inherited eye disease; affected by breed-typical MOD; or affected by another certifiable hereditary disease were all homozygous for the reference allele (S3 Table).

## Discussion

This study has identified a missense variant associated with MOD in the OES. Four of the seventeen cases are heterozygous for the variant inferring that MOD in the OES has a dominant mode of inheritance. The MOD phenotype of cases within this study is clinically different to a previous report of the condition in this breed [6]. This is the first variant to be reported for MOD in the dog and appears to segregate exclusively in this breed. The variant is a SNP located in a collagen type XI gene (*COL11A1)* in which pathogenic variants are known to cause syndromic conditions in humans; Stickler Syndrome Type II being the most analogous to the OES MOD phenotype described here [13, 20]. The clinical presentation of MOD in the OES in this study shares similar phenotypes with previous reports of the condition in other breeds, such as cataract, microphakia and retinal dysplasia [1–5]. A notable difference, however, is evinced in the globe abnormality, which in the current study was observed to be macrophthalmic in a number of dogs homozygous for the *COL11A1* variant and was reported on histopathology. This is antithetical to the microphthalmic globes reported in the aforementioned canine studies of multiple ocular anomalies, including the OES [6]. It is possible that two types of MOD exist in this breed and further research is warranted. Macrophthalmos in the OES, similar to the OSD-affected Northern Inuit dog, does not appear to be caused by elevated intraocular pressure (IOP) as it occurs despite normal readings. Abnormal collagen fibril architecture is a possible factor contributing to or causal for the occurrence [38, 39].

The cataracts in two MOD cases were designated as HC type and suspected in one, as shown in Table 3, and 89% of HC diagnosed OES were either carriers or homozygous for the *COL11A1* variant. This appears to support Barnett’s observations in UK OES (1978). According to the European College of Veterinary Ophthalmologists, the characterisation of HC in the OES breed is bilateral asymmetrical cortical opacities [9]. This description has a significant phenotypic overlap with the clinical characteristics of cataract in Stickler syndrome patients [40]. As with MOD-affected OES, cataract is a common phenotype in Stickler syndrome affected humans, and as with MOD OES the cataract type can vary. However, most patients assessed in a study by Seery and colleagues [40] had a ‘distinctive’ type that manifests as either peripheral, cortical, dense opacities or cortical punctate flecks. It is feasible that there is a different genetic variant causal for congenital HC not associated with MOD, segregating within the OES breed as one OES diagnosed at 0.16 years with congenital HC was found to be homozygous for the *COL11A1* reference allele. WGS analysis may aid in the molecular diagnosis of this case.

Collagen type XI is a heterotrimeric construct of α chains encoded by the *COL11A1*, *COL11A2* and *COL2A1* genes, with *COL11A1* expressed in ocular and connective tissue [20, 41, 42]. Differentiation between STL1 and STL2 can be made by the causal genetic variants for each type, which occur in the collagen type genes *COL2A1* and *COL11A1*, respectively. Inheritance of *COL2A1* variants is exclusively dominant with causal variants for the ocular-only subtype typically located within the alternatively spliced exon 2 [13]. STL2 genetic variants usually have a dominant mode of inheritance, although both recessively inherited and biallelic variants in *COL11A1* have been reported that result in a severe loss of hearing phenotype [43, 44]. Collagen type XI is highly expressed in the vitreous [45] and in this tissue it is encoded by *COL11A1*, *COL11A2* and *COL5A2*, which is a splice variant of *COL2A1* [46]. Collagen XI isoforms are denoted as ‘minor’ fibrils as they are the smallest component of the collagen fibre relative to other collagen types within the assembly. However, despite having a minor presence within the fibre, they have a major role in determining overall fibril diameter by regulating the number of nucleation events [13, 47]. Collagen fibril diameters vary in accordance with the quantity of collagen type XI present in differing ocular tissue types, low levels in the sclera result in a large fibril diameter and conversely in the vitreous, higher type XI relative quantities produce a narrower fibril diameter [45]. Examination of the vitreous in Stickler syndrome patients is used to differentiate between the type I and II and is described as ‘membranous’ in STL1 and ‘beaded’ in STL2 patients [46, 48]. The beaded vitreopathy observed in STL2 is reportedly due to irregular and thickened vitreous lamellae with pathogenic variants in *COL11A1*, a likely cause for the malformation [13]. Ocular ultrasound of MOD OES cases showed hyperechogenic strands/hyperechoic membranes, which suggests a similar form of vitreal pathogenesis in these dogs.

Vitreal syneresis was reported in OSD-affected breeds [15–17] and is the probable causative factor for subsequent retinal detachment. The vitreous architecture in Stickler syndrome is a factor that causes an increased risk of retinal detachment, which is estimated to be up to 65% higher than in any other condition where retinal detachment is prevalent [49]. Of the MOD OES cases reported to have a vitreopathy phenotype, the retinal detachment was observed in several cases at the initial appointment and later at re-examination. Pigment deposits in the anterior vitreous have also been suggested as a predictor of retinal detachment [50], an abnormality noted in an OES found to be heterozygous for the *COL11A1* variant. Abnormality of the vitreous was not described in all cases where retinal detachment occurred, very likely because not all cases underwent an ultrasound examination and it is possible that this phenotype was missed on the ophthalmic investigation; or it was not considered as a significant finding on ocular ultrasound to be recorded/reported. A histopathological exam revealed a loss of normal architecture of the iridocorneal angle which may be due to secondary chronic glaucoma or compatible with goniodysgenesis [51]. Although gonioscopy was normal in this MOD OES case on initial exam, progressive changes of iridocorneal angle are described in some breeds [52–54].

The MOD candidate variant is located at p.1592 of the *COL11A1* gene which is located in the C-terminus (also referred to as the COLFI domain) of the protein. Fibril assembly is initiated and controlled by the C-terminus, the region where the correct α fibril chains are selected and folded [55, 56]. The ability of strands to form either homotrimers or heterotrimers is conferred by the number of cysteine residues present within the COLFI domain, according to DiChiara and colleagues [55]. COLFI domains containing seven cysteine residues can assemble with dissimilar fibril strands and those with eight are restricted to forming homotrimers. In both the canine and human collagen type XI, *COL11A1* is the only one of the three constituent genes that contain seven cysteine residues in the COLFI domain with *COL2A1 and COL11A2* having eight. The MOD variant is located 26 amino acids upstream from the first cysteine residue. The importance of the cysteine residues in forming inter-fibril bonds is such that disruption of amino acid sequences at or near their location is likely to be pathogenic [55]. Bourhis *et al*. (2012) note that in the results of 46 missense mutations in the COLFI domains of five differing α chains (*COL11A1* was not included), trimerization was either inhibited, resulting in haploinsufficiency (in heterozygotes) or produced mutant assemblies that contained both wild type and mutant strands [57]. In STL2-affected humans haploinsufficiency procures a milder phenotype than observed in individuals with mutant type XI trimers, who exhibit a dominant negative phenotype [13, 14]. The *COL11A1* gene is alternatively spliced, thus allowing it to generate tissue-specific proteins [58]. In many STL2 cases it is missplicing due to a pathogenic genetic variant that is causal for the disease [13]. The number of missplicing events and therefore the proportion of incorrect transcripts produced are postulated to explain the variation in phenotypes between individuals, who share the same genetic variant [58, 59]. Furthermore, mosaicism due to *COL11A1* splice site variants has also been reported, and in one case, resulted in an almost imperceptible phenotype in the father of an STL2-affected child [60]. This variation in phenotypes renders a correct diagnosis of Stickler Syndrome challenging [13, 14, 46, 58–61]. Functional analysis of the variant would provide a greater depth of information as to the mode of pathogenicity on the protein within the MOD-affected OES. However, owing to a lack of ocular tissue this type of investigation was not possible.

*In-silico* analyses with some software tools suggest that the OES *COL11A1* variant results in an elevated density of exonic splice enhancers in conjunction with a reduction of silencers. Further research would be required to ascertain if there is any effect on splicing *in-vivo* and the levels of aberrant transcripts translated. What is evident, however, as manifested in human Stickler syndrome cases, is that there is an inter-individual variation of clinical characteristics in OES MOD cases which makes the classification of an overall phenotype rather complex. Our original approach to designate an OES as a MOD case was a confirmation of cataract and at least one other core phenotype as listed in Table 1. This led to the exclusion of samples provided with suspected MOD, and those excluded were listed in ‘Other ocular abnormalities’ (Table 3) with retinal detachment, retinal folds and pinpoint lenticular opacities. All aforementioned individuals carried the variant, with the OES with retinal detachment being homozygous. We cannot formally exclude the possibility that the disease is not fully penetrant in the OES, with 10 dogs confirmed to be clear of inherited eye disease heterozygous for the variant. However, Case 14 (Table 1) illustrates that the disease can have very late onset. Furthermore, at diagnosis Case 14 was still visual, the cataracts immature and abnormalities despite being numerous were described as mild or subtle. Furthermore, there is a formal possibility that the variant is in linkage disequilibrium with what may be the true causative variant for the condition. The variant in this instance would have be structural as a simple variant would have been detected during our WGS filtering process. However, when considering the in-silico predicted pathogenic effects (Section 3.2) the c.1775T>C variant is deemed to be highly damaging.

The ocular-only subgroup of Stickler syndrome has to date only been reported to be caused by variants located in the *COL2A1* gene, largely as a consequence of missplicing of exon 2, which is excluded in the protein in mature cartilage but included within ocular tissue [13, 20, 59]. *COL11A1* is also an alternatively spliced gene, however, pathogenic variants in humans result in additional systemic anomalies that can include cleft palate, deafness, hypermobility and arthropathy [13, 20]. Three OES, one homozygous and two heterozygous for the *COL11A1* variant were diagnosed with hip dysplasia. However, this was not a trait examined in this study and may be inconsequential to the *COL11A1* variant. Additionally, we received anecdotal reports of brachygnathia in three MOD OES cases. Two cases were included in this study and were homozygous for the MOD variant (S4 Table) but the third was reported as personal communication from a veterinary ophthalmologist. During an ophthalmic consultation, the latter OES MOD case was found to have an abnormal occlusion with a slight elongation of the mandible, however no x-rays were performed to confirm this.

In conclusion, WGS of a MOD-affected OES has identified a strong candidate missense variant in *COL11A1* within a gene region essential for the correct configuration of Type XI collagen. Segregation of the variant within the MOD-affected cohort revealed a dominant mode of inheritance and potentially a more severe presentation of the phenotype in the homozygous dogs. With a significant overlap of phenotypes, MOD in the OES appears to represent a canine form of STL2 and therefore may be used as a canine model for the disease. As yet, it is unknown whether the variant results in an ocular-only type or whether there are additional systemic anomalies. The discovery of this MOD associated variant will enable breeders to incorporate a DNA test within their breeding strategy to help reduce the incidence of this condition.

## Supporting information

S1 Table Phenotype keywords used in VarElect NGS phenotyper. (DOCX)

S2 Table Primers for Sanger sequencing of filtered variants in the MOD OES case. (DOCX)

S3 Table Control dogs of different breeds and health status genotyped for the *COL11A1* variant. (DOCX)

S4 Table Phenotypes of multiocular defect affected Old English Sheepdogs observed during initial consultation and any follow up examinations. (DOCX)

S1 Appendix NCBI multiple amino acid sequence alignment of 201 mammals. (DOCX)

S2 Appendix UMD Predictor-pro pathogenicity prediction of the *COL11A1* variant in the human. (DOCX)
