# Supplementary material for: Multiocular defect in the Old English Sheepdog: A canine form of Stickler syndrome type II associated with a missense variant in the collagen-type gene COL11A1
Source: PLoS One. 2023 Dec 28;18(12):e0295851. doi: 10.1371/journal.pone.0295851 (PMC10754463; doi:10.1371/journal.pone.0295851)
Supplement: S1 Table — (DOCX) [file pone.0295851.s001.docx]

| Lens |
| --- |
| Optical disc |
| Microphthalmia |
| Cataract |
| Congenital Cataract |
| Persistent pupillary membrane |
| Macrophthalmos |
| Coloboma |
| Enlarged globe |
| Globe |
| Microphakia |
| Dysgenesis of the iris stroma |
| Vitreous |
| Vitreal degeneration |
| Retinal dysplasia |
| Retina |
| Retinal detachment |
| Hypoplasia |
| Multiocular defects |
| Lens coloboma |
| Lens luxation |
| Keratitis |

**S1 Table**. Phenotype keywords used in Varelect NGS Phenotyper.
