# Supplementary material for: Multiocular defect in the Old English Sheepdog: A canine form of Stickler syndrome type II associated with a missense variant in the collagen-type gene COL11A1
Source: PLoS One. 2023 Dec 28;18(12):e0295851. doi: 10.1371/journal.pone.0295851 (PMC10754463; doi:10.1371/journal.pone.0295851)
Supplement: S2 Table — (DOCX) [file pone.0295851.s002.docx]

| **Gene** | **Forward Primer** | **Reverse Primer** | **Size (bp)** | **Private to Case** |
| --- | --- | --- | --- | --- |
| CDH5 | GGCTAAGGGACACTCACCTTC | ACGGTCAAGTTTGACAAGCAG | 498 | Yes |
| TCAF2 | CCCTGGTCTATCTGGTGACAA | GTCCACTCACCCAGCTTGTAG | 490 | Yes |
| COL4A2 | CGGAACATGTAGCTCTTCCAA | GAGTCCCAGACTAACCCGGTA | 292 | Yes |
| FLNA | GGTGTCACCCCTCAACAGAT | ATGTGCTCGTCACCCCACT | 281 | Yes |
| CDH4 | CTACGACTCCCTGCTGGTCTT | GGACCCCCTAACACACCACTA | 267 | Yes |
| ACAA1 | GGCACCCCACAGAACACC | GCCAGGTAAGCTTTTCTTTCTCAC | 300 | Yes |
| MYO5A | GTGGGGAGAAATAGGTCTCGT | TTTGCTGAGAGGTCACATTCC | 276 | Yes |
| NUP98 | GATTCCGGAACAGAGGAGAGT | CCCACCAATAATGCCATGTAG | 237 | Yes |
| SZT2 | AGCTGCGAATCCTCAGATG | AGCTGCGAATCCTCAGATG | 248 | Yes |
| MED1 | GTGTTTGGAGCGTTTTGTGAT | AGCCACCCAAAGTCAACACTA | 259 | Yes |
| SLC22A16 | TTTACAGGAGCTGGCCCTATT | ATCTCGTGGTGGTGTTTGTCT | 299 | Yes |
| CMYA5 | AGAGGGGTTTTATTGGTGTGG | TGACATGGAGGCCTTATCAAC | 497 | Yes |
| PYGM | CCCAATGACTTCAACCTCAAA | GACACAAAGGAGCCAACCATA | 473 | Yes |
| FBLN7 | CCAAGACCATCTCCTTCCATT | AAGAGTGTCAGCACTGCCCTA | 480 | Yes |
| TNXB | ACAGGAGGGGCCAGTGTAG | CTACGAGGGAGCGGACTGT | 298 | Yes |
| SEMA5A | GTTGGAGAGAGCCCTGTCTTT | CAGTACCGCATCTCGATTCTC | 282 | Yes |
| COL11A1 | GAAAATAGGAAACACATTGAAAAGC | GCAAGGATTCTCTGAAGCAGAT | 571 | Yes |
| IFT172 | AACACTCCGGAGATACCCACT | AGTGGGAAGCTCTTCTCCTTG | 544 | In DBVDC |
| PKHD | TGGTGGAAGCATACAATCCTC | TAGGAGGCAAGCTGATTTTCA | 468 | In DBVDC |
| CCDC47 | TTTACCTTCACGGTCAAATGG | CCAAAAAGTTCCGACTCAACA | 507 | In DBVDC |
| RCN2* | GCCGTGCCGCTGATTGGG | CTGCAGCCCTTCTCACCGAC | 205 | In DBVDC |
| LHCGR  (flagged as ENSCAFG002695) | TAATGTCTCCAGGCTGCATCT | CTGCTGCTGATGGAGGTTAAG | 526 | In DBVDC |

**S2 Table**. Primers for Sanger sequencing of filtered variants in the MOD OES case.
