# Supplementary material for: Multiocular defect in the Old English Sheepdog: A canine form of Stickler syndrome type II associated with a missense variant in the collagen-type gene COL11A1
Source: PLoS One. 2023 Dec 28;18(12):e0295851. doi: 10.1371/journal.pone.0295851 (PMC10754463; doi:10.1371/journal.pone.0295851)
Supplement: S3 Table — (DOCX) [file pone.0295851.s003.docx]

| **Breed** | **Number** | **Disease Status** | **Genotype** |
| --- | --- | --- | --- |
| Polish Lowland Sheepdog | 1 | Affected, Retinal Pigment Epithelial Dystrophy | Homozygous Reference (T/T) |
| Border Collie | 1 | Hereditary Cataract | Homozygous Reference (T/T) |
| Labrador Retriever | 1 | Progressive Retinal Atrophy | Homozygous Reference (T/T) |
| Flat Coated Retriever | 1 | Primary Closed Angle Glaucoma | Homozygous Reference (T/T) |
| English Bull Terrier | 1 | Primary Lens Luxation | Homozygous Reference (T/T) |
| Finnish Lapphund | 1 | Primary Closed Angle Glaucoma | Homozygous Reference (T/T) |
| Tibetan Spaniel | 1 | Progressive Retinal Atrophy | Homozygous Reference (T/T) |
| Beagle | 1 | Steroid Responsive Meningitis | Homozygous Reference (T/T) |
| Cocker Spaniel | 1 | Clear of inherited eye disease (9 yrs) | Homozygous Reference (T/T) |
| English Springer Spaniel | 3 | MOD Case | Homozygous Reference (T/T) |
| Cocker Spaniel | 1 | MOD Case | Homozygous Reference (T/T) |
| Labrador Retriever | 1 | MOD Case | Homozygous Reference (T/T) |

**S3 Table.** Control dogs of different breeds and health status genotyped for the *COL11A1* variant.
