# Supplementary material for: Multiocular defect in the Old English Sheepdog: A canine form of Stickler syndrome type II associated with a missense variant in the collagen-type gene COL11A1
Source: PLoS One. 2023 Dec 28;18(12):e0295851. doi: 10.1371/journal.pone.0295851 (PMC10754463; doi:10.1371/journal.pone.0295851)
Supplement: S4 Table — (DOCX) [file pone.0295851.s004.docx]

**S4 Table**. Phenotypes of multiocular defect affected Old English Sheepdogs observed during initial consultation and any follow up examinations (if applicable).

OD—right eye, OS—left eye, OU—both eyes; + = positive,— = negative; unless marked with *, lesions were present in both eyes; VR – vitreoretinal

1 M –male, MN–male neutered, FN–female neutered

2 ERG–electroretinography

3 OUS–ocular ultrasound

4 Follow up—period between first evidence of eye problem and last seen by veterinary surgeon; LIU–lens induced uveitis

5 BID–twice daily; TID – three times daily
