# Supplementary material for: Multiocular defect in the Old English Sheepdog: A canine form of Stickler syndrome type II associated with a missense variant in the collagen-type gene COL11A1
Source: PLoS One. 2023 Dec 28;18(12):e0295851. doi: 10.1371/journal.pone.0295851 (PMC10754463; doi:10.1371/journal.pone.0295851)
Supplement: S1 Appendix — (DOCX) [file pone.0295851.s005.docx]

**S5 Appendix.** NCBI multiple amino acid sequence alignment of 201 mammals (22 are predicted sequences). The sequence contains the location of the p.1592 *COL11A1* variant (highlighted) and is conserved across 200 of 201 species. There is no alignment in this region for *Carlito syrichta.*
