# Supplementary material for: Multiocular defect in the Old English Sheepdog: A canine form of Stickler syndrome type II associated with a missense variant in the collagen-type gene COL11A1
Source: PLoS One. 2023 Dec 28;18(12):e0295851. doi: 10.1371/journal.pone.0295851 (PMC10754463; doi:10.1371/journal.pone.0295851)
Supplement: S2 Appendix — (DOCX) [file pone.0295851.s006.docx]

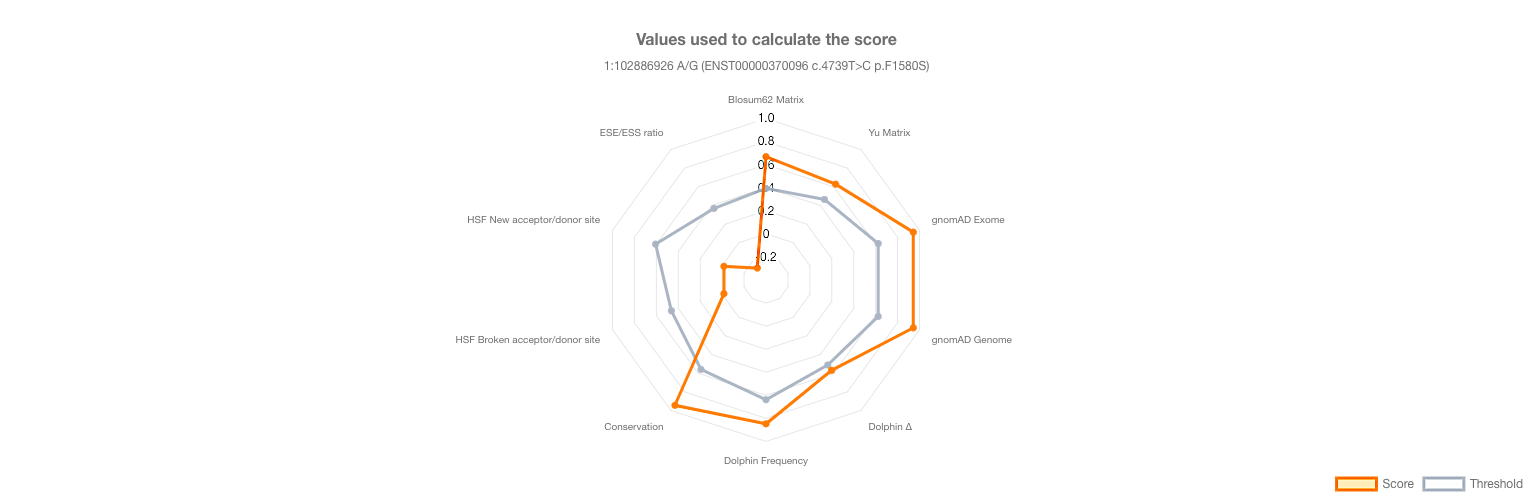

**
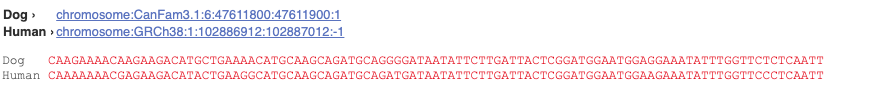
**

**S2 Appendix.** UMD Predictor-pro pathogenicity prediction of the *COL11A1* variant in the human. Genomic Position in canfam3.1 Chr6:47611886 and Chr1: 102886926 in GRCh38 in the human (Alignment from Ensembl illustrated above).
